# C-Reactive Protein Levels and Gadolinium-Enhancing Lesions Are Associated With the Degree of Depressive Symptoms in Newly Diagnosed Multiple Sclerosis

**DOI:** 10.3389/fneur.2021.719088

**Published:** 2021-10-26

**Authors:** Yavor Yalachkov, Victoria Anschuetz, Jasmin Jakob, Martin A. Schaller-Paule, Jan Hendrik Schaefer, Annemarie Reilaender, Lucie Friedauer, Marion Behrens, Christian Foerch

**Affiliations:** ^1^Department of Neurology, University Hospital Frankfurt, Frankfurt, Germany; ^2^Department of Neurology, Universitätsmedizin Mainz, Mainz, Germany

**Keywords:** multiple sclerosis, depression, inflammation, C-reactive protein, gadolinium enhancing lesion

## Abstract

**Background:** Inflammation is essential for the pathogenesis of multiple sclerosis (MS). While the immune system contribution to the development of neurological symptoms has been intensively studied, inflammatory biomarkers for mental symptoms such as depression are poorly understood in the context of MS. Here, we test if depression correlates with peripheral and central inflammation markers in MS patients as soon as the diagnosis is established.

**Methods:** Forty-four patients were newly diagnosed with relapsing-remitting MS, primary progressive MS or clinically isolated syndrome. Age, gender, EDSS, C-reactive protein (CRP), albumin, white blood cells count in cerebrospinal fluid (CSF WBC), presence of gadolinium enhanced lesions (GE) on T1-weighted images and total number of typical MS lesion locations were included in linear regression models to predict Beck Depression Inventory (BDI) score and the depression dimension of the Symptoms Checklist 90-Revised (SCL90RD).

**Results:** CRP elevation and GE predicted significantly BDI (CRP: *p* = 0.007; GE: *p* = 0.019) and SCL90RD (CRP: *p* = 0.004; GE: *p* = 0.049). The combination of both factors resulted in more pronounced depressive symptoms (*p* = 0.04). CSF WBC and EDSS as well as the other variables were not correlated with depressive symptoms.

**Conclusions:** CRP elevation and GE are associated with depressive symptoms in newly diagnosed MS patients. These markers can be used to identify MS patients exhibiting a high risk for the development of depressive symptoms in early phases of the disease.

## Introduction

Depression has a higher prevalence among multiple sclerosis (MS) patients as compared to non-MS-subjects (1) and is one of the most common comorbidities in MS (2, 3). It has a detrimental impact on patients' quality of life (4) as well as employment outcome (5) and is associated with an increased risk of disability worsening (6), resulting in adverse long term outcome (7).

Studies showing that physical disability predicts depression in MS (8) suggest that neurological deficits accumulate over time and lead to depressive symptoms. However, depression has been reported also for early MS and clinically isolated syndrome (CIS) patients (9–12), where physical disability is mostly mild or even non-existent. These observations indicate the existence of other mechanisms for the development of depression in MS.

One factor which has been shown to play a key role for the emergence of depressive symptoms in MS independent of disability is inflammation in the central nervous system (CNS). MS patients with an acute relapse have higher depression scores compared to patients in remission (13). Furthermore, tumor necrosis factor-α (TNF- α), interleukin-1β (IL-1β), and interleukin-6 (IL-6) measured in the cerebrospinal fluid of MS patients correlate with depression scores (13, 14).

On the other hand, major depression (MDD) studies stress the importance of peripheral inflammation markers. Thus, proinflammatory cytokines and molecules such as TNF-α, IL-6, interleukin 1 (IL-1), soluble interleukin 2-receptor (sIL-2R), and C-reactive protein (CRP) measured in serum are increased in patients with depression as compared to healthy subjects (15–18). Similar findings have been reported for MS: CRP levels are elevated in patients with as compared to those without a relapse and correlate with depression severity (19). Furthermore, increased IL-6 as well as decreased interleukin-4 (IL-4) and albumin in serum discriminate MS patients with from those without depression (20).

Treating depression in MS as early as possible and targeting its inflammatory mechanisms adequately require simultaneous investigations of peripheral and central markers of inflammation as early as the MS diagnosis is established. Such studies are largely lacking and it is not known whether both peripheral and central inflammatory processes are linked to depressive symptoms in newly diagnosed MS. We hypothesized that serological, CSF laboratory and CNS imaging markers of inflammation in patients with initial diagnosis of CIS, relapsing-remitting MS (RRMS) or primary progressive MS (PPMS) correlate with their depression scores.

## Materials and Methods

The study was approved by the ethics committee of University Hospital Frankfurt and carried out in accordance with The Code of Ethics of the World Medical Association (Declaration of Helsinki) for experiments involving humans. Written informed consent was obtained from all subjects. Patients were referred to the Department of Neurology at the University Hospital Frankfurt between 2017 and 2019 due to suspected demyelinating CNS disease either based on a clinical observation or on MRI imaging results. All patients underwent a neurological examination, laboratory tests, lumbar puncture, and MRI imaging as a part of a well-established diagnostic work-up based on the current guidelines of the German Neurological Society as well as the Competence Network Multiple Sclerosis. Patients were screened for eligibility and agreed to participate in the study and undergo additional measurements including Beck Depression Inventory (BDI) (21) and Symptom Checklist-90-R (22). BDI was used to assess depressive symptoms. As a validation of the results, additionally the Symptom Checklist-90-R (22) was applied and its depression dimension (SCL90RD) was entered into further analysis. Participants were included only if the diagnostic work-up resulted in the diagnosis of a relapsing-remitting multiple sclerosis (RRMS), primary progressive multiple sclerosis (PPMS) or clinically isolated syndrome (CIS) according to the 2010 revised McDonald criteria (23). The first participants were measured 2017 before the latest revisions of the McDonald criteria were officially published (24). At this time, we had already diagnosed and included several patients based on the McDonald 2010 criteria, therefore decided to keep these eligibility criteria unchanged. Exclusion criteria were diagnosis of secondary progressive multiple sclerosis or diagnosis of concurring neurological disease as a result of the diagnostic work-up (*n* = 4), determinable cause of an infection (*n* = 2), insufficient knowledge of German language to fill out the questionnaires or refusal to participate (*n* < 10).

Patients were interviewed and examined by a neurologist. Their degree of physical disability was estimated with the help of the Kurtzke Expanded Disability Status Scale (EDSS) (25). C-reactive protein (CRP, mg/dl) and albumin (g/l) were measured in serum as markers of peripheral inflammation. If multiple measurements were available, the ones with the greatest proximity in time to the questionnaires were used. CRP was considered elevated if it was ≥0.5 mg/dl. In this case, physical examination and laboratory testing were performed, including auscultation of the lungs and urinalysis. If a determinable cause of infection was found, the participant was excluded from further analysis. White blood cells count in CSF (CSF WBC/μl) was used as a marker of central inflammation. Gadolinium enhancement (GE) on T1-weighted imaging in at least one of the following: cerebral, spinal or orbit MRI imaging was another marker of CNS inflammation. We computed for each participant also the total number of typical MS lesion locations (juxtacortical, periventricular, infratentorial, and spinal) with T2- or FLAIR-hyperintense lesions (“MS lesions,” number of typical locations with MS lesions varying between 1 and 4).

Some patients were treated with intravenous methylprednisolone (IVMP) for their neurological symptoms. CSF acquisition was done always prior to IVMP. To consider any possible influence of IVMP on depressive symptoms, patients were grouped according to the time between IVMP and BDI/SCL90RD: the first group did not receive any IVMP or the interval between IVMP and BDI/SCL90RD was >14 days (ΔIVMP-BDI/SCL90RD > 14 days), while the second group was treated with IVMP within 14 days before the BDI measurement (ΔIVMP-BDI/SCL90RD ≤ 14 d). To consider the influence of IVMP on GE, patients were grouped according to the time between IVMP and MRI measurement: the first group did not receive any IVMP or the interval between IVMP and MRI was >14 days (ΔIVMP-MRI > 14 d), while the second group was treated with IVMP within 14 days before MRI (ΔIVMP-MRI ≤ 14 d).

First, a multiple linear regression was computed with BDI as dependent variable. Age, gender, EDSS, CRP elevation, albumin, CSF WBC, GE, and MS lesions were employed as independent variables. Next, a second multiple linear regression with the same independent variables but with SCL90RD as dependent variable was computed to validate our first results. Linear relationship was verified by inspecting the respective scatter plots of independent and dependent variables. Normal distribution of the residuals was verified by inspecting the respective P-P plots. There was no evidence of multicollinearity (highest correlation *r* = −0.417, lowest Tolerance statistics = 0.625, and highest VIF statistic = 1.6). Independence of residuals was tested by determining Durbin–Watson statistic (Durbin–Watson = 2.5 for BDI and 2.25 for SCL90RD). Homoscedasticity was tested by inspecting a graph plot of the standardized values predicted by the respective model against the standardized residuals. The lack of influential cases biasing the model was verified by determining Cook's Distance (all Cook's Distance values < 1).

To understand better the interaction between peripheral and central inflammation, we computed two further univariate general linear models with BDI and SCL90RD as dependent variables and the two markers which contributed significantly to the variance in the linear regression analyses (CRP elevation and GE, see section Results) as independent variables.

To assess the effects of steroid treatment on depressive symptoms, a multivariate general linear model was computed with BDI and SCL90RD as dependent variables and ΔIVMP-BDI/SCL90RD as an independent variable. To assess the effects of steroid treatment on acute inflammation seen in MRI, a Fisher's exact test was computed with GE and ΔIVMP-MRI.

Finally, we tested in an exploratory analysis whether disease type and fatigue measurements might affect our findings. Fatigue measurements were available from clinical routine diagnostics (The Fatigue Scale for Motor and Cognitive Functions, FMSC) (26) for some but not all subjects (*n* = 40). We computed an additional multiple linear regression like the ones mentioned above but with “disease type” (RRMS, CIS, or PPMS) and “FSMC total score” as additional independent variables. Since MS disease type can affect not only depression but also inflammation, we also computed a Fisher's exact test with disease type and CRP elevation.

## Results

One patient was excluded due to the combination of CRP elevation and an acute venous leg ulcer and another one due to a still active urinary tract infection in antibiotic treatment at the time of measurement. The data of the remaining 44 subjects were entered into the analysis. Their demographic data is shown in Table 1. The medical history of the patients is reported in Table 2.

**Table 1:** Descriptive statistics of the studied sample.

		***n***	**x**	**SD**
Gender	Female	32		
	Male	12		
Diagnosis	RRMS	31		
	CIS	8		
	PPMS	5		
CRP	Elevated	6		
	Normal	38		
MS lesion locations	1	7		
	2	11		
	3	12		
	4	14		
GE + in at least one	Yes	30		
MRI imaging	No	14		
ΔIVMP-MRI	>14 days	32		
	≤ 14 days	12		
ΔIVMP-BDI	>14 days	18		
	≤ 14 days	26		
Age (years)			35.68	10.63
EDSS			2.02	1.28
CSF WBC (/μl)			11.09	11.78
Albumin (g/l)			44.95	4.60
BDI			6.77	6.25
SCL90RD			0.64	0.62

*n, number of patients in the respective category; x, mean value; SD, standard deviation; RRMS, relapsing-remitting multiple sclerosis; CIS, clinically isolated syndrome; PPMS, primary progressive multiple sclerosis; CRP, C-reactive protein (elevated: ≥0.50 mg/dl; normal <0.50 mg/dl); MS lesion locations, total number of typical MS lesion locations (juxtacortical, periventricular, infratentorial, and spinal) per subject with T2- or FLAIR-hyperintense lesions; GE +, gadolinium enhancement in T1-weighted imaging; ΔIVMP-MRI > 14 days, no steroid treatment or the interval between IVMP and MRI was >14 days; ΔIVMP-MRI ≤ 14 days, interval between IVMP and MRI ≤ 14 days; ΔIVMP-BDI/SCL90RD > 14 days, no steroid treatment or the interval between IVMP and BDI/SCL90RD was > 14 days; ΔIVMP-BDI/SCL90RD ≤ 14 days, interval between IVMP and BDI/SCL90RD ≤ 14 days; EDSS, Expanded Disability Status Scale; CSF WBC, white blood cells count in the cerebrospinal fluid; BDI, Beck Depression Inventory; SCL90RD, depression dimension of the Symptoms Checklist-90-Revised*.

**Table 2:** Past medical history and medications reported in the sample.

**Medical history**	**Medication**
Arterial hypertension (x 1)	Estradiol (x 1)
Bronchial asthma (x 1)	Levothyroxine (x 9)
Carpal tunnel syndrome (x 1)	Oral contraceptives (x 2)
Chronic back pain (x 1)	Orthomol (x 1)
Coccygeal fistula (x 1)	Progesterone (x 1)
Depression (x 1)	Salbutamol (x 1)
Epilepsy (x 1)	Sumatriptan (x 2)
Factor V Leiden (x 1)	Vitamin D (x 1)
Hashimoto thyroiditis (x 3)	
Hypothyreosis (x 5)	
Lactose intolerance (x 1)	
Migraine (x 4)	
Obesity (x 1)	
Spondylarthritis (x 1)	
Status post autoimmune thyroiditis (x 1)	
Status post Bell's palsy (x 1)	
Status post benign tumor resection (x 1)	
Status post herpes zoster infection (x 2)	
Status post Lyme disease (x 1)	
Status post other fracture (x 1)	
Status post pelvic fracture (x 1)	
Status post sinusitis (x 1)	
Status post strabismus correction (x 1)	
Status post tonsillectomy (x 1)	
Status post type C gastritis type (x 1)	
Status post vestibular neuritis (x 1)	

The multiple linear regression model with BDI as dependent variable was significant (*R* = 0.603, *R*^2^ = 0.364, adjusted *R*^2^ = 0.218, *F* = 2.502, df1 = 8, df2 = 35, and *p* = 0.029). Two independent variables contributed significantly to the model: CRP elevation (ß = 0.431, *t* = 2.87, and *p* = 0.007) and GE (ß = 0.362, *t* = 2.46, and *p* = 0.019, see Table 3).

**Table 3:** Details from the two multiple linear regressions.

			**95% CI for B**				
	**B**	**SE B**	**LB**	**UB**	**β**	***t***	***p***
**BDI**							
Age	0.02	0.10	−0.19	0.22	0.03	0.16	n.s.
Gender	2.58	2.10	−1.68	6.85	0.19	1.23	n.s.
EDSS	0.46	0.71	−0.98	1.90	0.09	0.65	n.s.
CRP elevation	7.77	2.70	2.28	13.26	0.43	2.87	0.007
Albumin	0.26	0.22	−0.20	0.71	0.19	1.14	n.s.
CSF WBC	0.10	0.09	−0.08	0.27	0.18	1.14	n.s.
GE	4.81	1.95	0.84	8.78	0.36	2.46	0.019
MS lesion locations	−0.92	0.88	−2.70	0.86	−0.16	−1.05	n.s.
**SCL90RD**							
Age	0.00	0.01	−0.02	0.02	−0.02	0.02	n.s.
Gender	0.12	0.21	−0.30	0.54	−0.30	0.54	n.s.
EDSS	0.10	0.07	−0.04	0.25	−0.04	0.25	n.s.
CRP elevation	0.82	0.27	0.28	1.37	0.28	1.37	0.004
Albumin	0.01	0.02	−0.03	0.06	−0.03	0.06	n.s.
CSF WBC	0.00	0.01	−0.01	0.02	−0.01	0.02	n.s.
GE	0.39	0.19	0.00	0.78	0.00	0.78	0.049
MS lesion locations	−0.17	0.09	−0.35	0.00	−0.35	0.00	n.s.

*BDI, Beck Depression Inventory (dependent variable); SCL90RD, depression dimension of the Symptoms Checklist-90-Revised (dependent variable); B, unstandardized B coefficient; SE B, standard error of the B coefficient; 95% CI, 95% confidence interval; LB, lower bound; UB, upper bound; β, standardized beta coefficient; t, t-value; p, p-value; n.s., not significant; EDSS, Expanded Disability Status Scale; CRP elevation, C-reactive protein elevation (elevated: ≥ 0.50 mg/dl; normal < 0.50 mg/dl); CSF WBC, white blood cells count in the cerebrospinal fluid; GE +, gadolinium enhancement in T1-weighted imaging*.

The multiple linear regression model with SCL90RD as a dependent variable was significant (*R* = 0.609, *R*^2^ = 0.371, adjusted *R*^2^ = 0.227, *F* = 2.583, df1 = 8, df2 = 35, and *p* = 0.025). Two independent variables contributed significantly to the model: CRP elevation (ß = 0.461, *t* = 3.09, and *p* = 0.004) and GE (ß = 0.298, *t* = 2.04, and *p* = 0.049, see Table 3). The other variables, CSF WBC, EDSS, age, gender, albumin, and total number of typical MS lesion locations, were not predictive for depressive symptoms.

Both CRP elevation and GE were entered as independent variables in two further general linear models in order to exploratory illustrate the interaction between peripheral and central inflammation markers in the context of depression in newly diagnosed MS. With regard to BDI, CRP elevation and GE exhibited significant main effects (CRP elevation: *F* = 7.113, *p* = 0.011; GE: *F* = 9.087, *p* = 0.004) and the interaction between the two factors was significant, too (CRP elevation × GE: *F* = 4.492, *p* = 0.04). Elevated CRP values or presence of gadolinium enhancement on the T1-weighted MR imaging resulted into higher BDI scores (CPR normal: x = 5.7, SD = 4.4; CRP elevated: x = 13.8, SD = 11.2; GE –: x = 4.4, SD = 3.5; GE +: x = 7.9, SD = 6.9), while the combination of both elevated CRP and gadolinium enhancement had a particularly strong impact on the depression score (x = 18, SD = 11.5). Vice versa, the majority of patients with normal CRP levels and lack of GE in MRI exhibited normal BDI scores. However, either GE in MRI or CRP elevation were associated with BDI scores corresponding on average to a minimal or mild depression, while patients with both CRP elevation and GE in MRI had BDI scores which corresponded to at least a moderate depression (21). A similar pattern was revealed with regard to SCL90RD. Here, however, only CRP elevation reached significance (*F* = 8.428, *p* = 0.006), while GE (*F* = 3.737, *p* > 0.05) and the interaction between the both factors (*F* = 1.202, *p* > 0.05) failed to do so.

The multivariate general linear model did not reveal any significant effects of the time interval between IVMP treatment and the depression questionnaires on the BDI/SCL90RD scores (*F* = 0.121, *p* > 0.05). The Fisher's exact test revealed no significant effects of the time interval between IVMP treatment and MRI imaging on the presence of GE (*p* > 0.05).

The exploratory linear regression with BDI as dependent variable and with additional independent variables (disease type and FSMC total score additionally to age, gender, EDSS, CRP elevation, albumin, CSF WBC, GE, and MS lesions) was significant (*R* = 0.696, *R*^2^ = 0.485, adjusted *R*^2^ = 0.307, *F* = 2.726, df1 = 10, df2 = 29, and *p* = 0.017). Like in the initial analysis, two independent variables contributed significantly to the model: CRP elevation (ß = 0.373, *t* = 2.312, and *p* = 0.028) and GE (ß = 0.381, *t* = 2.522, and *p* = 0.017). Disease type and fatigue measurements did not reach significance (*p* > 0.05). The Fisher's exact test revealed no significant effects of the disease type on CRP elevation (*p* > 0.05).

## Discussion

In the current study, depressive symptoms of patients with newly diagnosed RRMS, CIS, or PPMS were linked to peripheral and central markers of inflammation. More precisely, patients with elevated CRP or presence of GE on T1-weighted MRI-imaging were more likely to score higher on depression scales BDI and SCL90RD. Further analysis showed that the link between CRP/GE and depression is even more pronounced if both inflammation markers are simultaneously present.

The role of CRP elevation has been highlighted so far mainly by studies with MDD patients (15–17, 27). Recent findings suggest that inflammation contributes to the emergence of depression, since elevated CRP predicts the subsequent development of depressive symptoms (28). The pathophysiological mechanisms are complex and involve different pathways: peripherally released signals including CRP and cytokines cause an inflammatory response in the CNS, altering production, metabolism and transport of mood-related neurotransmitters and affecting neuronal growth and survival (27). Further mechanisms, including oxidative stress, cytokine-induced glutamate dysregulating and excitotoxicity as well as maladaptive hypothalamic-pituitary-adrenal (HPA) axis functioning have been suggested (19, 20, 27). CRP elevation might be distinctly relevant for therapeutic decisions, too. Thus, a treatment with the monoclonal antibody infliximab, an TNF-α antagonist, resulted in a greater reduction of depressive symptoms in a subset of medication resistant MDD patients with high baseline CRP levels (29). Furthermore CRP is among the inflammatory signal molecules with the strongest relationships with depression (15, 16) and is easily obtained and analyzed in hospital laboratories, rendering it readily utilizable in a clinical context (27).

The source of CRP elevation in MS patients seems even more complex than in MDD. CRP is higher during MS relapses and associated with EDSS, predictive for later progression and decreasing during interferon beta 1a therapy (30–32). Therefore, peripheral inflammation is probably linked to general disease activity in MS, too. However, interactions with environmental factors might lead to an increased risk for the emergence of depression as has been shown for MDD: adults diagnosed with MDD who have a history of early maltreatment exhibit higher CRP levels than those without such a history (33). Thus, MS-related inflammation coupled with external events which also boost inflammatory responses might be crucial for the development of depressive symptoms in early MS.

CNS inflammation markers might contribute further to understanding the role of the immune system in the context of depression. Generally, GE on T1-weighted MRI imaging indicates a disrupted blood-brain barrier and characterizes active MS lesions (34). In our sample, the presence of active lesions was significantly associated with the degree of depressive symptoms. This extends earlier findings (13, 35), confirming the link between CNS inflammatory lesions and mood disorders in MS and validating it with regard to newly diagnosed patients. While it can be argued that active lesions induce neurological impairment and that depressive symptoms arise only as a result from the newly emerging physical disability, our findings do not support this notion. Indeed, the degree of depressive symptoms did not correlate with EDSS. Furthermore, GE was present in a multitude of CNS regions (e.g., spinal, infratentorial, or optic nerve lesions), which implies that mood alterations in the context of newly diagnosed MS occur also without direct lesions to the limbic system. Rather, depressive symptoms may be triggered by more general inflammatory mechanisms. Moreover, the total number of typical MS lesion locations with T2- or FLAIR-hyperintense lesions was not correlated to the mood symptoms, suggesting that the degree of disease activity in MS is not determining for the emergence of depression in newly diagnosed patients. However, since all our patients were newly diagnosed and were thus exposed to a novel, stressful situation (new symptoms, initial uncertainty, major diagnosis, etc.), a more complex interplay between CNS inflammation and external factors seems possible here, too. Interestingly, major negative stressful events predict increased risk for GE in MRI imaging in MS patients (36). Thus, an interaction between inflammatory MS-related processes and confrontation with a spectrum of novel, potentially stressful events such as physical disability and major diagnosis may have contributed to the development of depressive symptoms in our sample.

CRP elevation and GE did not explain the whole variance of depression in our sample. Similarly, it has been estimated that only 47% of depression patients whose scores are above the clinical threshold had a CRP level ≥ 0.3 mg/dl and only 29% had a CRP level ≥ 0.5 g/dl (27, 37), suggesting an essential but perhaps not sufficient role of peripheral inflammation for the emergence of depression and a more pronounced relevance for a subset of depressed individuals. Interestingly, we demonstrated an interaction between peripheral and central inflammation leading to more pronounced depression scores, which points at a possible mutually augmenting effect of peripheral and central inflammatory agents. The correlational nature of our results does not allow us to imply causation but one possible, hypothetical mechanism would be a common pathway of inflammation starting in the periphery and resulting in blood-brain barrier breakdown, unfolding inflammation in CNS and triggering depressive symptoms in early MS. Similar interactions between peripheral and central processes have been shown for MDD, where high CRP levels are associated with gray matter volume reductions (38) as well as with reduced functional connectivity in a widely-distributed brain network (39, 40). It would be highly relevant to follow-up whether the depressive symptoms persist in the further course of MS and if the initial peripheral and central inflammation parameters are predictive for their future development.

MS disease type has previously been shown to be relevant for depression and inflammation (4, 41, 42). Similarly, fatigue might affect or co-occur with depression (42, 43). However, in our exploratory linear regression analysis, disease type and fatigue were not associated with BDI, while CRP and GE presence were again significant predictors for depressive symptoms. Furthermore, CRP elevation did not differ between the three investigated disease types. The small sample size as well as the fact that we did not include SPMS patients could have contributed to these findings. An interesting yet purely tentative notion refers to the fact that we studied newly diagnosed patients only. Thus, the pathophysiological underpinnings of inflammation and depression might be very similar across different MS disease forms during early disease stages and the effect of fatigue on depression might grow in later stages of the disease (41).

There are several limitations to our study. First, we employed a cross-sectional design, which does not allow us to determine a causal relationship between the measured inflammation and degree of depressive symptoms. For this purpose, a prospective design would be necessary. Second, the sample size was moderate. We included only patients with newly established diagnosis, thus leaving out effects of immunomodulatory therapy, accumulated physical disability or other disease-related confounding variables. However, this reduced the power of the study and may have contributed to the lack of significant effects regarding inflammation markers such as albumin and CSF WBC. Especially with regard to CSF WBC the results are surprising since CSF inflammatory markers such as TNF-α and IL-1β measured in the cerebrospinal fluid of treatment-naive MS patients correlate with depression scores (13, 14). The insufficient power of the study due to our conservative inclusion criteria would explain the lack of positive findings regarding CSF. Another possible explanation is that in this study we focused on the CSF WBC, while leaving out other, possibly more specific inflammatory markers such as TNF-α and IL-1β. Second, measurements were done as a part of a diagnostic work-up tailored for the clinical routine, which did not allow us to standardize all the parameters (e.g., MRI scanner, time of lumbar puncture and blood sample collection, IVMP treatment, etc.). While we did not observe any effects of IVMP treatment on the primary outcome parameters, a prospective, standardized approach with predetermined parameters would have increased the methodological quality and validity of our results. This is, however, a common challenge of studies in the clinical setting, when patients without an established diagnosis are referred to the emergency department at night and cannot wait with the IVMP treatment because of pronounced neurological disability. Similarly, it is difficult to determine the time interval between communicating the diagnosis to the patients and the measurements of depression and inflammation markers, since many patients are referred for a diagnostic work-up with a suspected diagnosis which they already know of. However, standardizing the measurements of this time interval as far as possible would limit the influence of external factors such as diagnosis communication on subjects' mood. Finally, we employed only self-assessment instruments such as BDI and SCL90R. Using clinical interviews which are available for depressive symptoms would increase the validity of the results.

Despite the above-mentioned limitations, our study contributes several important findings to the scientific debate. First, we demonstrate that the relationship between inflammation and depression is seen not only in patients with a prolonged disease history but also in newly diagnosed MS when the disease is presumably in its initial stages. Furthermore, the reported findings are not affected by immunomodulatory and symptomatic therapy. Moreover, contrary to the common expectation that depressive symptoms are seen in MS only when neurological impairment is strongly pronounced, we demonstrate that even in a sample of newly diagnosed MS patients with little to none accumulated physical disability (average EDSS = 2.02, SD = 1.28) there is a link between inflammation and depression. Studies focusing on adults with a major depressive disorder seldomly employ MRI with contrast-agent or perform a cerebrospinal fluid analysis. As illustrated in this study, investigating interactions between inflammation and depression in newly diagnosed MS patients, in whom those examinations are part of the diagnostic work-up, seems particularly promising. Of course, the chronic inflammation typical for MS remains an important issue to tackle when interpreting the data. Prospective studies investigating the link between inflammation and depression simultaneously in both newly diagnosed MS and MDD patients in early stages of the disease might be essential in addressing this issue.

In our study we demonstrated that peripheral (CRP) and central (GE on T1-weighted MRI imaging) inflammation markers are associated with the degree of depressive symptoms in newly diagnosed patients with RRMS, PPMS, and CIS. Understanding which MS patients exhibit higher risk for depressive symptoms would facilitate the development of eligible therapeutic options, targeting depression-relevant inflammatory pathways.

## Data Availability Statement

The raw data supporting the conclusions of this article will be made available by the authors, without undue reservation.

## Ethics Statement

The study was reviewed and approved by Ethics Committee of University Hospital Frankfurt. The patients/participants provided their written informed consent to participate in this study.

## Author Contributions

YY: drafting/revision of the manuscript for content, including medical writing for content, major role in the acquisition of data, study concept or design, and analysis or interpretation of data. VA and MB: major role in the acquisition of data and analysis or interpretation of data. JJ, JS, AR, and LF: major role in the acquisition of data. MS-P: drafting/revision of the manuscript for content, including medical writing for content, and major role in the acquisition of data. CF: drafting/revision of the manuscript for content, including medical writing for content, and study concept or design. All authors contributed to the article and approved the submitted version.

## Conflict of Interest

YY has been supported by travel grants from Novartis and Sanofi Genzyme, has received an honorarium for active participation in an advisory board by Sanofi Genzyme as well as speaking honoraria by Roche and Sanofi Genzyme. CF reports speaker honoraria and honoraria for participating in advisory boards from Alexion, Novartis, Teva, Merck, Sanofi-Genzyme, and Roche and he received research support from Novartis and Sanofi-Genzyme. The remaining authors declare that the research was conducted in the absence of any commercial or financial relationships that could be construed as a potential conflict of interest.

## Publisher's Note

All claims expressed in this article are solely those of the authors and do not necessarily represent those of their affiliated organizations, or those of the publisher, the editors and the reviewers. Any product that may be evaluated in this article, or claim that may be made by its manufacturer, is not guaranteed or endorsed by the publisher.
